# The Current Trends of Biosensors in Tissue Engineering

**DOI:** 10.3390/bios10080088

**Published:** 2020-08-03

**Authors:** Yi-Chen Ethan Li, I-Chi Lee

**Affiliations:** 1 Department of Chemical Engineering, Feng Chia University, Taichung 40724, Taiwan; 2 Department of Biomedical Engineering and Environmental Sciences, National Tsing Hua University, Hsinchu 300044, Taiwan

**Keywords:** biosensors, tissue engineering, organs-on-chips, label-free biosensors, cell-based biosensors, tissue/organ-based biosensors

## Abstract

Biosensors constitute selective, sensitive, and rapid tools for disease diagnosis in tissue engineering applications. Compared to standard enzyme-linked immunosorbent assay (ELISA) analytical technology, biosensors provide a strategy to real-time and on-site monitor micro biophysiological signals via a combination of biological, chemical, and physical technologies. This review summarizes the recent and significant advances made in various biosensor technologies for different applications of biological and biomedical interest, especially on tissue engineering applications. Different fabrication techniques utilized for tissue engineering purposes, such as computer numeric control (CNC), photolithographic, casting, and 3D printing technologies are also discussed. Key developments in the cell/tissue-based biosensors, biomolecular sensing strategies, and the expansion of several biochip approaches such as organs-on-chips, paper based-biochips, and flexible biosensors are available. Cell polarity and cell behaviors such as proliferation, differentiation, stimulation response, and metabolism detection are included. Biosensors for diagnosing tissue disease modes such as brain, heart, lung, and liver systems and for bioimaging are discussed. Finally, we discuss the challenges faced by current biosensing techniques and highlight future prospects of biosensors for tissue engineering applications.

## 1. Introduction

Biosensors encompassing a wide range of knowledge and technologies, including chemistry, physics, and biology, are emerging as the next-generation detection technology. By combining the disciplinary technologies, biosensors offer a versatile platform for analytical applications in food, environmental, microbiology, public health, and biomedical science. In general, a biosensor system consists of receptors of biological samples, transducers, and display systems to detect the results through electrical, chemical, or optical components and then convert the recognition event into a measurable signal [1]. Through these components, biosensors aim to measure minuscule signals from a small number of samples, providing a useful procedure with stability and high sensitivity for rapid analysis without the need of specialized laboratory training and skills. The first biosensor was designed by Clark and Lyons in the early 1960s. Their design utilized glucose measurement by detecting electrochemical properties through an electrode with immobilized glucose oxidase [2]. Since then, incredible progress has been made [3] in both fabrication techniques and tissue engineering applications of biosensors with innovative approaches involving electrochemistry, photolithography, and nanotechnology to impedance [4]. Currently, bioreceptor parts in biosensors have been widely designed with various functions for detection of toxicants, proteins/genes, cells or tissue behaviors [5,6]. In clinical applications, molecule-based biosensor is a fundamental type of biosensors to detect the cytokines, proteins, or genes from patients.

The molecule-labeled biosensors use specific biochemical reactions mediated by isolated enzymes, receptors, antigens and antibodies, DNA and ion channels as the recognition elements [7]. In particularly, sensing cellular secretions in microfluidic biochips involves immobilizing bio-recognition elements such as antibodies or aptamers in close to cells [8,9]. The coupling of biological components with high-affinity biomolecules allows the high sensitivity and selectivity of a range of analytes which provide the main superiority of molecular-based biosensors [10]. To analyze targeting molecules, enzymes, antibodies, or specific genomic probes such as nucleic acids are bound on the sensor surface by using chemical graft or physical adsorption methods. Then, the change of electrochemical, optical, calorimetric, or piezoelectric signals from transducers could be transferred as electrical signals for display in short times [11]. For example, Arya and co-workers fabricated an interleukin-2 (IL-2) biosensor by using a 4-fluoro-3-nitrophenyl (FNP) as a linker to graft the IL-2 antibody on a gold electrode surface. Via the interactions between the antibody and the antigen, the results of cyclic voltammetry (CV) confirmed the sensing function of this anti-IL-2 immobilized gold electrode [12]. Furthermore, Chen et al. developed aptamer-based mesoporous silica nanoparticles (MSN) as a biosensor to execute a control release system [13]. They conjugated double strain DNA in the pore of MSN through a click chemistry method to cap FITC fluorescent dyes encapsulated in MSN. After thrombin specifically recognizing DNA aptamers on the MSN, the encapsulated FITC fluorescent dyes could be released from the pore of MSN to enhance the fluorescent intensity, indicating that biosensors also enable to be used as a stimuli-responsive control release system. Moreover, physical properties of material surfaces also provide a strategy as a biosensor to recognize different molecules. Previous study has been reported that Au/Ag-graphene quantum dots as an adsorption agent to modify the surface of a glassy carbon electrode enable to adsorb prostate-specific antigen (PSA) through the high affinity between proteins and Au/Ag [14]. This immunosensor offers an ultra-sensitivity to quantitate the level of PSA in serum, facilitating the early diagnosis of prostate cancer disease.

In addition to molecule-based biosensors, cell- or tissue-based biosensors provide an opening for the development of in situ monitoring cell techniques in the past decade [15,16,17]. These types of label-free biosensors provide the advantages to observe cellular and tissue responses from cellular systems by measuring phenotypic responses with temporal resolutions [18]. Basically, cell-based biosensors contain living cells, bioactive substrates, and transducers. Through the bioactivity of substrates, cells cultured on the biosensors could express their specific cellular polarity or biomarkers. After treatment of biochemical or pharmaceutical agents, the effects of these agents on cells could be recorded via the change of cellular polarity or physiological parameters such as permeability of cell membrane or ligand expression of cells on biosensors [15]. Compared with cell-based biosensors, although molecule-based biosensors provide a high selectivity for detection, short useable lifetime, and expensive isolation cost of bioactive identifying molecules limit the applications of this type of biosensor. Therefore, the concept of cell-based biosensors offers a real-time strategy to develop a rapid and biomimetic system for diagnosis of diseases. To obtain more precise diagnosis results, biosensors are moved forward from cell level to tissue level. In term of tissue-based biosensors, multi-cellular culture and organoids are usually used as biomimetic constructs towards native tissues. Via the use of multi-cellular culture or organoids, it enables the engineering of complex 3D tissue-like constructs which recapture the features and functions of native tissues in a 3D physiological environment. Thus, state-of-the-art in vitro 3D biomimetic constructs endow the tissue-based biosensors with potential biosensing strategies for predicting, monitoring, and diagnosing pharmaceutical effects on human outcomes. Currently, the biomimetic properties of in vitro 3D constructs make them attractive for use as a novel tissue-based biosensor system, called an organ-on-a-chip platform. Through connection with a dynamic control system, the integrated system could mimic physiological environments such as dynamic fluid flow or force in human body. For example, Bavli and co-workers developed a tissue-based biosensor by using a liver organoid and particle-type oxygen sensors [19]. This system provides a real-time liver-like response to track the mitochondrial dysfunction after the treatment of drugs.

Here, we examine current trends of biosensors in biomedical science field, including the fabrication technologies, types, and versatile applications in different tissues of biosensors, to highlight how biosensors are utilized in tissue engineering studies. With the review, we expect to contribute a general view which could allow scientists to understand the needs and principle of biosensors for the advanced biomedical applications, further combine their techniques to develop biosensors with potentially novel functions for the evaluation, prediction, diagnosis, and discovery of human diseases.

## 2. Current Technology for Fabrication of Biosensors

As above mention, biosensors are generally divided into two main categories, label-based or label-free biosensors which include biochips, paper-based biosensors, nanoparticles, and flexible biosensors. In the past decade, many techniques, such as photolithography, computer numerical control (CNC), and casting, have been well defined to fabricate these two types of biosensors. In general, biosensors are fabricated through not only one of these methods but also combination of two more methods, indicating the current techniques offer a versatile strategy to design simple or complex sensing systems.

Photolithography is a technique widely used for patterning cells and proteins in tissue engineering and biosensor applications. Photolithography is the process of transferring the geometric shapes on a mask to a silicon or a glass surface through UV exposure [20]. The steps involved in the photolithographic process are pattern designing, surface cleaning, pattern printing on a photomask, pattern transferring from the mask to the substrate, soft baking, polymer pouring, exposure, and hard-baking [21]. For biosensors applications, microelectrodes with different sizes and shapes can also be patterned on surface of biochips with by photolithography techniques, allowing for the production of transducer components with very small dimensions [22].

A CNC machining technique has been developed from 1940s. Until now, CNC machining is a technique fully developed and widely used for making various sensors. A CNC maneuverable platform consists of controlling multiple axes (i.e., xyz axes) and different cutting tools such as drills, waterjet, and saws etc. Furthermore, scientists could generate a desired design of their biosensors by using graphical computer-aided design (CAD). Afterwards, a combination of CAD and a CNC platform provides a maneuverable tool to rapid machine the substrates with patterns or shapes following the user’s design. For example, Pires and co-workers fabricated a nitrite biosensor by using the CNC technique to mill microchannels on a PMMA chip. The photoactive organic compounds, polythieno[3,4-b]thiophene/benzodithiophene and (6,6)-phenyl-C71-butyric-acid methyl-ester, as a thin layer were integrated with the PMMA chip. Then, the nitrite existed in the samples could be assayed by detecting the change of signal-to-noise ratio of fluorometric intensity when the sample fluid were conducted through the CNC-milled channels [23]. This CNC-machined biosensor shows its potential applications for preventing the brown blood disease in fish. Today, some CNC platforms at the factory level have been augmented with four to six axes compared with traditional three-axes type CNC platforms, enabling the production of detecting systems with 3D fine structures. Usually, in biosensor systems, the CNC technique is used to machine poly(methyl methacrylate) (PMMA) substrates to fabricate large scale biosensing systems.

Biochips with biosensors require pattern cells in specific locations to create biomimetic structures. The size, shape, and surface topology of pattern and the surface modification are the factors to affect the cell attachment and cell behaviors. Sometimes, the abovementioned photolithographic technology is combined with the CNC technique to integrate a biochip offering a microfluidic flow as a dynamic biosensing system. In previous literature, several bio-recognition elements have been patterned on biochips with interdigitated electrode arrays to perform as DNA sensors, immune-sensors or enzymatic sensors [24,25].

**Figure 1:**
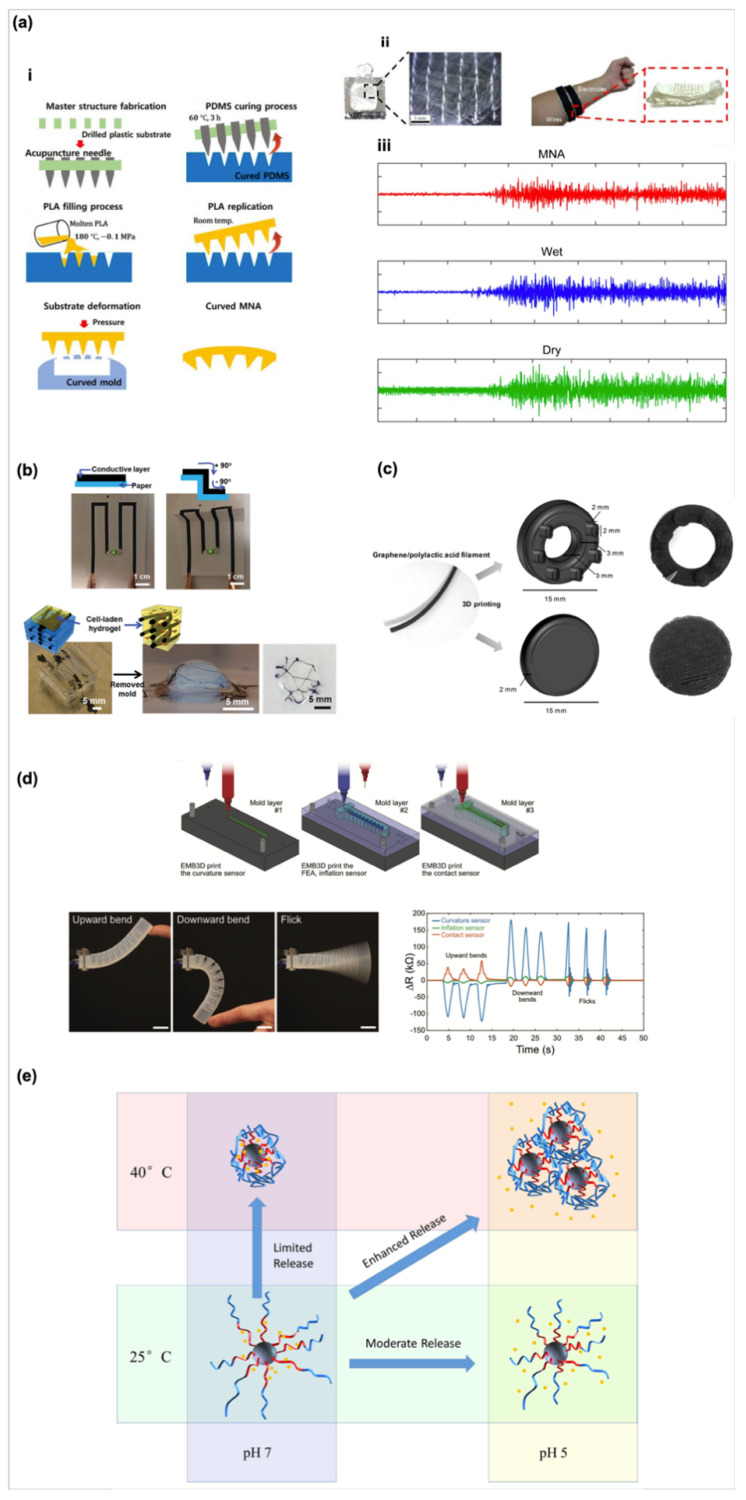
(**a**) Fabrication process of a curved microneedle by using an acupuncture needle as a mater structure to make a negative polydimethylsiloxane (PDMS) model. Then, filling melt poly(lactic acid( (PLA) into the negative PDMS model, a curved PLA microneedle can be obtained through the casting process. (Reprinted with permission from [30] Copyright (2015) MDPI publishing.); (**b**) The light-emitting diode (LED) light connected with a carbon nanotube (CNT) ink-printed circuit on a paper before and after folding. Moreover, the CNT ink-printed fibers could be encapsulated in a hydrogel and used as a 3D biosensor for the detection of cardiac electroactivity. (Reprinted with permission from [31] Copyright (2016) John Wiley & Sons publishing.); (**c**) The morphology and dimensions of a 3D printed ring shape graphene-based biosensor. (Reprinted with permission from [32] Copyright (2018) ACS publications.); (**d**) A 3D printed technique is used to fabricate a somatosensitive biosensor containing a dorsal sensor (layer 1), an actuator features and an inflation sensor (layer 2), and a contact sensor (layer 3). The resistance change could be recorded and be significant distinguished three type of resistance changed behaviors during the upward, downward, flicked movements of somatosensitive biosensor. (Reprinted with permission from [33] Copyright (2018) ACS publications.); (**e**) The sensing mechanism of silica/poly(methacrylic acid) (PMAA)- poly(N-isopropylacrylamide) (PNIPPAM) core-shell nanoparticles indicate that temperature and pH effects contribute to the nanoparticles with the enhanced release and moderate release behaviors, respectively. (Reprinted with permission from [34] Copyright (2017) ACS publications.).

Photolithography and CNC techniques endow biosensor systems with a versatile of small or large scale to detect the targeting physiological parameters. However, the existing issues restrict their applications from being suitable for all situations [26]. For example, the cleaning, exposing, and other manufacturing steps of the photolithographic process need the rush substrates as accessories such as organic solvents or high temperature [27], while the drill of the CNC machine also generates a high temperature during the working procedure. These issues easily destroy or change the polarity, roughness, and other surface properties of materials and cause the occurrence of unexpected and uncontrol side effects so that the quality of electrodes at the following patterning process may be affected [28]. In addition, some of substrates such as silicone or PMMA used in photolithography and CNC techniques may not be suitable for ligand/protein adsorption, modification, even in living cell culture, that restrict the feasibility of use of bioactive materials. To overcome these issues, an alternative strategy, casting, is developed for fabrication of biosensors. Soft lithography and casting technologies, provide a simple process to pattern the surface morphology of materials. Scientists could make a reverse mold through photolithography and CNC machining processes; then by casting the user-desired materials on the reverse mold enables to generate the desired patterns on casting materials after crosslink of the casting materials. A microneedle array electrode fabricated through the casting technique has been reported for continuous measurement in the dermal layers with minimal pain. For example, the metal microneedle array electrodes are functionalized by entrapping glucose oxidase in the electropolymerized polyphenol films for invasiveness, continuous monitoring and sensing [29]. Additionally, Kim and co-workers developed a PDMS band-based microneedle sensor by using two molds with negative needles and positive structures [30]. Then, this flexible microneedle sensor with a curved shape (Figure 1a) as a surface electromyography not only maintains the stable skin contact, but also enhances the intensity of signal measurement.

Soft or flexible biosensors attract significant interest as a new type of sensors. To make soft or flexible types of biosensors, many strategies have been developed in the past ten years. For example, Shin and co-worker used carbon nanotube (CNT), DNA, and Gelatin methacrylol (GelMA) as a conductive ink [31]. This CNT-based ink enables to pattern a 2D circuit on a cellulose paper. Interesting, the LED light could remain its brightness even the circuit bent under various curvature angles. In addition to the 2D paper-based sensor, they further immersed the CNT-based conductive fibers into hydrogels as a 3D conductive electronic to stimulate the electrophysiological signals of cardiac cells (Figure 1b). These techniques indicate directly patterning a conductive ink on a paper or in a hydrogel with a potential as a flexible biosensor for biomedical applications. In addition, transfer-printing technology and kirigami technology are widely used to fabricate biosensors [35,36]. The transfer-printing technology is a technique that the designed pattern is directly written on a donor substrate by using an ink. Consequently, a flexible stamp will pick up the pattern from donor substrate to a receiver. The advantage of this technique is that the designed pattern could be easily printed on the substrates with various shape or mechanical properties [35]. Sim et al. have reported a paper-based sensor which is fabricated by using the transfer-printing technology. They further confirm that this flexible paper-based sensor could fit curvilinear skin surface well and precisely measure the electromyography signal from skin [37]. The kirigami technology is usually a strategy to design flexible sensors by using laser or CNC machining processes to cut the substrates with a close-shape, planar, and rotational symmetric structure. This feature provides a way to make soft and flexible sensors with 3D mesostructures [38]. Therefore, Evke et al. designed a kirgami sheet with integrated strain gauges as a strain sensor [39]. This strain sensor could transform into a 3D mesostructured spring which could fit on joint well and represent the movement and position of body skeletons. These studies provide a novel concept to develop a soft, flexible, and wearable biosensors. Currently, 3D printing has emerged as a versatile technology that allows the fabrication of 3D constructs with high complexity at extremely high spatial precision and reproducibility. To date, a variety of different techniques for bioprinting have been proposed, such as extrusion, [40,41,42,43] injection, [44,45,46,47] magnetic, [48] and laser-based approaches [49,50,51]. To make 3D complex constructs, the additive manufacturing technique of 3D printing provides a rapid process to construct the user-desired structures by layer-by-layer stacking materials. Thus, 3D printing is a suitable candidate for fabricate various types of biosensors [52]. Palenzula and co-workers provide a proof-of-concept to make ring- and disk-shaped electrodes by printing a graphene/poly(lactic acid) ink for electrochemical sensing (Figure 1c) [32]. Through the results, it is confirmed that the printed electrodes maintain high sensitivity to sense ascorbic acid with a wide detection range from 10 to 500 μM. Moreover, Truby and co-workers developed an embedded printing technique to fabricate a somatosensitive actuator consisting of curvature, inflation, and contact sensors which can display the change of resistance during the upward/downward movements and flicks (Figure 1d) [33]. This report further showed that a sensory feedback from a soft robotic gripper assembled by printed somatosensitive actuators possesses the feasibility of 3D printing technique used for fabricating soft robotic biosensors.

Next, nanoparticles have been developed as another novel type of biosensors in therapeutic and diagnostic applications [53]. The nanoscale of nanoparticles endows with them with high surface areas which contribute more contact probability to nanoparticles for capturing the targeting analytes [54]. For example, magnetic nanoparticles are usually modified their surface function groups by grafting antibodies, peptides, nucleus acids [55]. Via the specific binding interactions between antigens and antibodies, the magnetic nanoparticles could rapidly sense the specific targeting analytes in a short time; thus, the nanoparticles could be easily collected under a magnetic field after capturing the analytes [56]. Furthermore, many nanoparticles such as gold- or graphene oxide-based nanoparticles offer stimuli-responsive functions as biosensors which are widely designed as control released systems. For example, silicone-based nanoparticles with poly(methacrylic acid) (PMAA) and poly(N-isopropylacrylamide) (PNIPAM) mixed shell provide dual pH-and thermo trigger properties as a control released cargo for cancer therapy applications (Figure 1e) [34]. The negative charged carboxylic groups on PMAA chains provide an electrostatic attraction to conjugate positively charge DOX under a neutral pH condition. Then, the protonation of carboxylic groups on PMAA occurs following the decrease of pH, results in the decrease of electrostatic interaction, and enhances the release of doxorubicin (DOX). Moreover, through the combination effect of hydrophilic and hydrophobic polymer chain transitions of pNIPAM polymers under different temperatures, nanoparticles with a PMAA/PNIPAM-mixed shell offer a synergistic effect on the release of DOX from cargo and the DOX uptake of cancer cells. This information provides an open view for designing nanoscale biosensors using nanoparticles.

## 3. The Applications of Biosensors in Tissue Engineering

Tissue engineering involves the principles of biological and engineering sciences toward the development of engineered tissue constructs as biological substitutes to replace, maintain, or repair lost functions of damaged tissues. [57] Tissue engineering strategies require integration of cell-cell interactions and cell-material interactions via incorporation of appropriate cellular and physical signals. Therefore, it is necessary to determine and monitor the cellular signals, cell behaviors, cellular responses, and cell functionalities. Recently, biosensors have been widely used in applications of tissue engineering. In particularly, the development of tissue engineering systems in microfluidic platforms with biosensors for sensing specific biological macromolecules and cellular behaviors with the miniaturized tissue constructs are popular. These miniaturized systems provide the important information for tissue engineering construct evaluation and physiological response prediction in rapidly, real-time, and low dose levels, through optical, electrical, electrochemical, and impedance sensing systems [58]. Here, we further discuss the essential functions of biosensors for various applications in tissue engineering field.

### 3.1. Biosensors for Cellular Applications

#### 3.1.1. Biosensors for Cell Polarity

Polarity probes or polarity-based biosensors are useful to investigate on biochemical processes which occur at membrane or subcellular levels. As reported, a polarity-sensitive annexin-based biosensor with switchable fluorescence states has been fabricated on the detection of cell apoptosis with live cell images [59]. This biosensor was applied on investigation of both in vitro and in vivo dynamic changes in neuron degeneration process.

#### 3.1.2. Biosensors for Cell Behaviors Such as Metabolism, the Proliferation/Differentiation of Stem Cells

**Figure 2:**
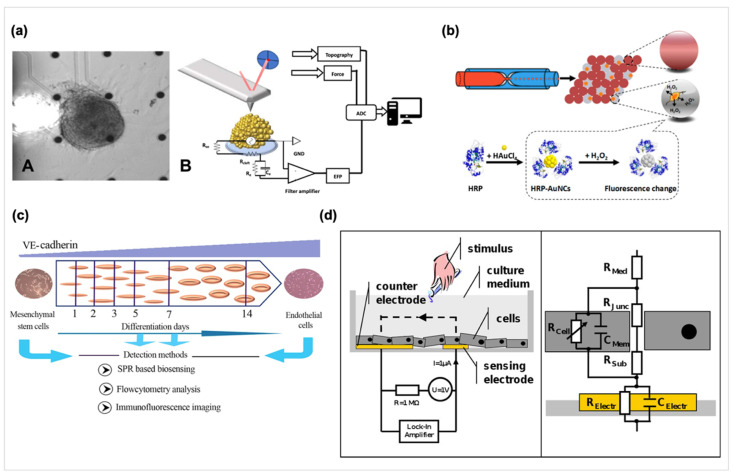
(**a**)-A: A beating cardiac cluster placed on a microelectrode arrays (MEA) sensing area. (**a**)-B: Schematic representation of the implemented setup (Reprinted with permission from [62] Copyright (2019) ELSEVIER publishing). (**b**) Schematic Illustration of Detecting Hydrogen Peroxide in the Single-Cell Encapsulated Droplets in Combination with horseradish peroxidase (HRP)-AuNCs (Reprinted with permission from [64] Copyright (2018) ACS publishing). Real case and schematic of the proposed electromechanical cell-based system. (**c**) Schematic of surface plasmon resonance (SPR) biosensor for vascular endothelial (VE)-cadherin expression for evaluating of human mesenchymal stem cells trans-differentiation to endothelial lineage (Reprinted with permission from [70] Copyright (2017) ELSEVIER publishing). (**d**) Schematic of the electric cell-substrate impedance sensing (ECIS) system and representative equivalent circuit for an adherent growing cells layer. Left: Cross section of an ECIS culture well. Right: ECIS measures the sum of all individual contributions to the impedance (Reprinted with permission from [74] Copyright (2014) MyJove Corporation publishing.

Biosensors for cellular applications focus on the fields of cell behaviors and cellular signals determination, disease modeling, and drug toxicity or bioactive molecular assessment. Thus, several approaches for transduction of cellular signals are performed, such as cell metabolism measurement, intracellular and extracellular potentials determination. Evaluation of electrophysiological properties of neural-related cells and cardiomyocytes is important for tissue engineering and microelectrode arrays (MEAs) is a candidate for measuring electrophysiological properties of cells. Chowdhury et al. considered that the contact electrogram is related with cellular action potential and should change in conjunction with each other during arrhythmogenesis. They developed a novel technique combining MEA recordings with optical mapping to simultaneously record the contact electrograms and action potentials [60]. Also, biomechanical measurements on cardiomyocytes for investigating the pathophysiological electro-mechanical coupling were done by using the stretcher device previously [61]. Further, Caluori et al. combined the MEA technology and atomic force microscope system for recording beating force of the cardiomyocytes cluster and the triggering electric events, as shown in Figure 2a [62].

Zhu et al. have also monitored the increased secretion of pre-loaded norepinephrine on a MEMS device via the expression of G protein coupled receptors from a fibroblast cell line as the basis for a living cell-based biosensor [9]. In another report, fluorescent microbead-based sensors were designed to detect the transforming growth factor-β1 and the hepatocyte growth factor secreted by primary hepatocytes diffuse through the hydrogel barrier into adjacent sensing channels [63]. Furthermore, some other devices are designed to measure the release of other important signaling molecules like hydrogen peroxide (H_2_O_2_) or Nitric oxide (NO) [64,65]. H_2_O_2_ affects immunity generation, cell migration, and cellular communications, and NO is an important gaseous messenger in the biological system. As shown in Figure 2b, the combination of a microfluidic approach with droplets and Au nanoclusters for sensing H_2_O_2_ secreted by a single cell and a poly(toluidine blue)-modified electrode has been constructed for the determination of nitric oxide, respectively. Furthermore, Visser et al. used inkjet printing techniques to print carbon MEAs on the extracellular matrix mimetic materials consisted with PDMS, gelatin, and several kinds of hydrogel to fabricate soft MEAs [66].

### 3.2. The Methods of Biosensors Detecting Cell-Related Analytes

#### 3.2.1. Label-Free Biosensors

Based on the abovementioned applications, the quantification of cell number, migration, or protein expression is generally used to detect the cellular behaviors under a specific culture condition. Conventionally, the measurement of cell number and viability can be generally realized by directly counting cells, detecting the viability such as the MTT test, or quantifying DNA content. However, these methods are labor-intensive, time-consuming processes, and involve cell sacrifice. Noninvasive and label free detecting methods provide the advantages no require to stain, fix, and fluorescent-labeled which are desirable in stem cell technology and tissue engineering applications.

#### 3.2.2. Surface Plasmon Resonance (SPR) Based Biosensors

SPR biosensors have emerged as a versatile biosensor recently which provides the advantages of high throughput, small sample volumes, and live cell analysis. SPR biosensors also allow for real-time label-free analysis of biomolecule interactions on functionalized surfaces based on their high sensitivity to the change of the tested objects’ refractive index [67]. A novel SPR-based biosensing apparatus allowing the detection of optical properties difference of the cell surface was designed and applied to evaluate the osteogenic differentiation of mesenchymal stem cells [68]. Also, the SPR biosensor has been used on monitoring the release of two clinically decisive cardiac biomarkers, fatty acid binding protein 3 and cardiac troponin T for cardiotoxicity effects evaluation on hESC-derived cardiomyocytes [69]. Further, as shown in Figure 2c, Fathi et al. developed an SPR sensing system with SPR signals rapidly and accurately response cell differentiation by detecting of vascular endothelial-cadherin expression which could sense the early stage of endothelial differentiation on day 3 [70].

#### 3.2.3. Impedance-Based Biosensors

Electric cell-substrate impedance sensing (ECIS) is an in vitro method to quantify the behavior of cells by measuring impedance, providing a real-time, label-free, non-contact, and non-destructive cellular analysis. ECIS provides an alternative or assistive method to conventional biological assays for tissue engineering and diagnostic applications. ECIS measures cells grown on a small area on single electrode surfaces via the high frequency AC impedance, as shown in Figure 2d [71,72,73,74]. The properties of the cultured cells attached onto the interdigitated electrodes can be measured through the change of electrical impedance of electrodes. In this situation, the capacitance depends upon the quality of cell-cell contact rather than that of cell-electrode contact. Measuring cellular impedance allows the automated study of cell attachment, growth, differentiation, and motility. Previous studies have used ECIS measurement to quantify cell behaviors in a confluent layer, involving cell motility, barrier functions, quality of cell-material and cell-cell adhesions, and quantification of cellular responses and wound healing to vasoactive stimuli [74]. An on-line continuous method based on ECIS was also developed to measure the cell growth and cytotoxicity of fibroblasts exposed to mercury chloride and 1,3,5-trinitrobenzene. It is considered that the response function of cells exposed to drug was derived to reflect the resistance variation as a result of cell adhesion, spreading, migration and cytotoxicity effect [75]. Moreover, Koo.Y. et al. used ECIS to create a wound by applying high current, and then real-time monitoring the healing process through the measurement of impedance [76]. Also, ECIS was used on measurement the kinetics of human melanoma cell invasion across human brain endothelium [77]. Moreover, Pierre et al. used ECIS on real-time monitoring stem cells differentiation by measured variations in the complex impedance Z* throughout induction of adipose stem cells differentiated into osteoblasts and adipocytes on multielectrode arrays. The detection parameters of an ECIS Zθ instrument are over a 62.5–64 kHz frequency range and 180 s per time for 17 days. They found that osteoblast and adipocyte lineages have distinct dielectric properties and the differences can be detected by impedance sensing and applied on monitoring adult stem cell differentiation [78]. Fleischer et al. has developed a system to compare the cardiomyopathies from iPSCs on 2D culture and 3D clusters by impedimetric and electrophysiological monitoring [79]. Another novel design using 3D self-rolled biosensor arrays with active field-effect transistors or passive microelectrodes was reported for the electrophysiological properties measurement of human cardiac spheroids in 3D [80].

### 3.3. Biosensors in Tissue/Organ and Their Derivative Disease Models

#### 3.3.1. Biosensors in Neural Disease Models

Biosensor-based systems contribute a convenient strategy to detect various signals from tissue, implying that biosensor-based systems possess potential applications for diagnosis of diseases. Traditional diagnosis of neurological diseases are time-consuming and inconvenient because it needs a doctor checking the symptoms from patient overcomes and exists a risk that around 40% of people can’t be estimated in some diseases such as Parkinson’s disease (PD) at early stage [81]. Neurological research is another field of investigation where cell-based biosensors have proven to be significant and the MEA technology is also the primary mode of determine neuronal circuits, physiology and abnormalities [82]. The MEA technique provides the advantages of non-invasive monitoring the electrophysiological activity of neurons for a long term culture, multi-site recording, and high-throughput screening [83]. The collective electrophysiological behavior of the neuronal network in terms of burst activity on MEA was applied on pharmaceutical agents testing. In addition, in order to analyze spreading depolarization, Lourenco et al. developed an innovative multimodal approach for metabolic, electric and hemodynamic measurements with neuronal activity [84].

**Figure 3:**
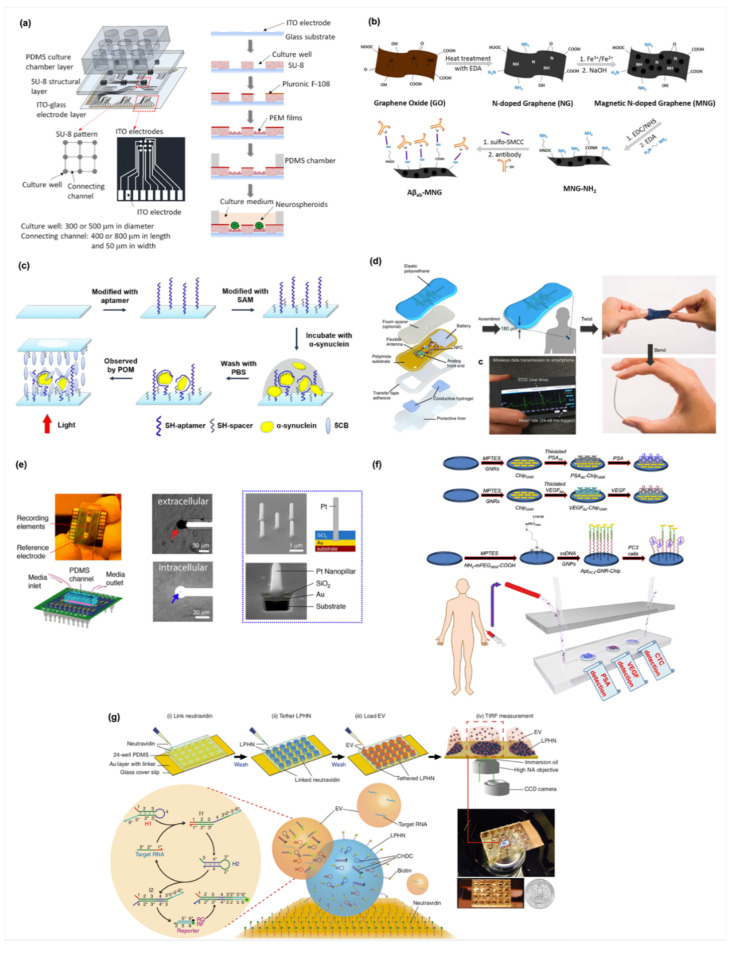
(**a**) Schematic illustrating the design and fabrication of a biochip by using the MEA technique. The patterned channels with Indium ion oxide (ITO)-electrodes provide a connected network enabling to guide the neurite outgrowth and measure the neurological signals through impedance signals. (Reprinted with permission from [85] Copyright (2018) ACS publications). (**b**) Illustrate the preparation of Aβ42-immobilized graphene-based biosensors. The magnetic graphene nanomaterials assembled on an Au electrode provide a re-usable biosensor for rapidly detect the Alzheimer’s disease-related biomarker. (Reprinted with permission from [92] Copyright (2016) Springer Nature Limited.) (**c**) Schematic illustrating the detection of a Parkinson’s disease (PD) biomarker (α-synuclein) through a liquid crystal biosensor. After the deoxyribonucleic acid (DNA) aptamer on the biosensor surface capturing the α-synuclein, the bright view of liquid crystal could be observed in the polarized microscope. (Reprinted with permission from [93] Copyright (2020) Royal Society of Chemistry) (**d**) Schematic images of a soft flexible cardiac biosensor. The real-time heart rate and electrocardiogram (ECG) waveforms from this biosensor could be transmitted to smart phone to display a visible signal on an app. Additionally, the flexible polyurethane substrate endows biosensor with an ability to deform under the mechanical twist and bend force. (Reprinted with permission from [94] Copyright (2018) Springer Nature Limited.) (**e**) A heart-on-a-chip was assembled by an electrode and PDMS channels for culturing cardiac cells. The nanopillars on Au electrodes could be as a transducer to puncture into cellular membrane for the activation ATP-dependent K^+^ channels and promotion of membrane repolarization. (Reprinted with permission from [95] Copyright (2020) ACS publications) (**f**) Illustration of a prostate cancer biosensor integrated a microfluidic chip and vascular endothelial growth factor (VEGF)- and prostate-specific antigen (PSA)-immobilized electrodes. The biochip offers a high sensitivity for the detection of prostate cancers and their circulating tumor cells and an easy quick method to analyze by using a UV-Vis absorbance within one hour. (Reprinted with permission from [96] Copyright (2017) Ivy spring International Publisher) (**g**) Schematic illustrating the principle and operation of the biochip by linking neutravidin with a tether lipid-polymer hybrid nanoparticle (LPHN) and loading extracellular vesicle (EV) on an Au layer. This technique enables to distinguish patients with early- or late-stage pancreatic cancers. (Reprinted with permission from [97] Copyright (2017) Springer Nature Limited.).

Based on MEA technique, our lab has developed a novel interdigitated microelectrode arrays which combines electric stimulation on the guidance of neurite outgrowth ITO-PEM microfluidic system and the neuronal network development on different patterns by an impedimetric monitoring system, as shown in Figure 3a [85]. The biochip was constructed with a PDMS culture chamber layer, an SU-8 structural layer, and an indium tin oxide (ITO)-glass detector layer, as illustrating in (Figure 3a) [85]. The bottom of each culture well contains an embedded ITO electrode. The second layer, the SU-8 structural layer, consists of a pattern of 3 × 3 arrays of culture wells. The culture wells and the connecting channels were designed to be 300 and 500 μm in diameter, and 400 and 800 μm in length, respectively. Then, the structural layer was treated with oxygen plasma and polyelectrolyte multilayer films (PEMs) to guide neural stem cells differentiation. The electrical connections of two neurospheroids were determined by measuring the impedance across two electrodes. Impedance measurements were conducted using an impedance analyzer. A potential of 0.1 Vrms was applied across the electrodes and the impedance data from 1 Hz to 10 kHz were collected for each measurement. Impedance at 1 kHz quantitatively described the linkage of neurites and was used to analyze the electrical connections of the neural network. Because cell membranes are electrically insulated, impedance measurements have been used to analyze cellular responses [86,87,88]. A significant difference was observed between unconnected and connected neurites. When the neurospheroids attached on the ITO electrodes without connecting neurites, the effective electrode surface area was reduced and led to an increase of the impedance value across the electrodes. In contrast, when the neurospheroids were connected by neurites, electrical connections were constructed and the impedance value across the electrodes was significantly reduced. A threshold of 40 kΩ was defined to determine the electrical connections of the neurospheroids. Neural communication and regeneration were investigated using not only immunocytochemistry but also electrical stimulations and recordings. Impedance measurements were conducted across two neurospheroids to provide quantitative evidence and validate the connections of the neural network. The development of organ on chips provide an alternative on drug discovery, tissue engineering, and biomaterials testing, and disease model. Biosensors combined biochips are promising tools and contribute significantly to these research fields. In particularly, integrated biosensor and biomimetic chip systems can monitor stem cells proliferation, early stage differentiation, neural network formation, electrophysiological properties, and stimulation response.

Furthermore, ultra-flexible and micro-electrocorticography arrays fabricated by glassy carbon electrodes have also been used to stimulate and record brain activity with low background noise [89]. For example, Xie and co-workers designed a microporous nanoelectronic probe to overcome the mechanical mismatch and the instability of traditional silicon- and metal-based microprobes used in brain tissue [90]. They fabricated a 3D microporous device integrated by wire-shape nanoelectrodes; this microporous probe offers an ultra-flexibility and an optimized neuron/probe interface to promote the integration with brain and record the action potential activity from somatosensory cortex in brain. Other applications where live cells-based devices have been applied on monitoring of endothelial barrier functions, live cell secretion, and molecules releasing [91]. Recently, Li et al. developed reversible electrode for rapidly diagnosis of Alzheimer’s disease by using magnetic graphene nanomaterials (Figure 3b) [92]. They conjugated the antibody of Alzheimer’s disease biomarker, Amyloid-beta peptide 1–42 (Aβ42), on a magnetic nitrogen-doped graphene (MNG). Then, an Alzheimer’s disease biosensor could be rapidly constructed by dropping the magnetic MNG immunocarriers on an Au electrode surface which has a tapping permeant magnet at the underside of electrode. Afterward, the used MNG biosensors could be removed and the Au electrode could be reproduced by switching of the tapped permeant magnet. Moreover, another biosensor with rapidly detection of PD has been reported by Yang and co-workers (Figure 3c) [93]. They generated a self-assembled monolayer (SAM) by grafting a DNA aptamer and an SH-spacer on the surface of substrate. After incubation with the PD biomarker (α-synuclein), the DNA aptamer provides a specific binding to capture α-synuclein protein, and the backfilled CH_3_(CH_2_)_9_SH (C_10_SH) and CH_3_(CH_2_)_15_SH (C_16_SH) mixture induce an aligned liquid crystal. Furthermore, the changed optical signals caused from the binding of α-synuclein could be easily recognized by using a microscope. Compared with traditional ELSA or Western blot methods, these methods offer novel, rapid, and easy strategies for the detection of neurological diseases.

#### 3.3.2. Biosensors in Cardiac Disease Models

According to the above applications, it clearly indicates that the measurement of physiological electrical signals is one of basic features of biosensors. Noticeably, bioelectrical activity is also an important myocardial function which can respond to the health of heart tissues. In general, the bioelectrical activity could be generated from the cardiomyocytes which are induced the change of action potential of cellular membrane; through the change of action potential, heart could be induced a synchronized pumping behavior via this organized electrical propagation [98]. A previous study reported that the change of oxygen level in tissues significantly interrupts the regular organized duration of action potential [99]. Therefore, a continuous electrocardiogram (ECG) monitoring technique provides a clinical standard method to detect the cardiac rhythm signal for diagnosing cardiac related diseases. To easily monitor cardiac rhythm signals, Lee et al. developed a small wearable flexible cardiac sensor which integrates an electrode, a near-field-communication chip, and a battery in polyurethane substrates [94]. The small cardiac biosensor offers a real-time visible signal that people could directly observe the change of cardiac rhythm signals on their smart phones (Figure 3d). In addition, Feiner and co-worker developed a degradable electronic scaffold as a cardiac patch. The gold electrodes are deposited on an electrospun albumin-fiber scaffold as passivation layer. Through this design, the flexible cardiac patch enables to sense the spontaneous contraction signal of cardiac cells and further provide an external electrical stimulation for regulate the contraction of cardiac cells [100]. The results indicate that the electronic albumin-fiber scaffold have a potential to use as a cardiac patch. Furthermore, a heart-on-a-chip system containing biosensors also contribute to a living heart cell system to mimic heart-related diseases. Liu and co-workers fabricated a Pt nanopillar array on an Au electrode (Figure 3e) [95]. Then, a PDMS microfluid channel was stacked on the Au electrode and cardiac cells were perfused and seeded on that to create a hypoxia-like heart disease environment such as myocardial infraction. Significantly, the biosensor displays narrow action potential signals consistent with a possible mechanism that oxygen-deficits enhance the activity of ATP-K channel and the repolarization of cellular membrane. This heart-on-a-chip type biosensor provides an in vitro disease model for investigating and understanding the effect of hypoxia on the electrophysiological behaviors of heart.

#### 3.3.3. Biosensors in Live/Lung and Immune Systems Disease Models

Similar to brain- and heart-on-a-chip systems, other types tissue models such as liver and lung are also designed with embedded biosensors to detect the functions of tissue-like living constructs. For example, mitochondrial dysfunction involves in the development of chemical or pharmaceutical toxicity. Balvi et al. used HepG2 cell-based liver organoids as a tissue model and cultured the organoids in a microfluidic device [19]. By real-time monitoring the metabolic function of liver organoids, it endows the microdevice as a biosensor with a feature to track the dynamic of mitochondrial dysfunction. Via sensing the oxidative phosphorylation of glycolysis or glutaminolysis, the liver organoid-based system permits to evaluate the safety and effect of drug concentration on mitochondrial damage. Besides, diagnosis or prognosis of immunosystem disease are also another essential topic in biosensor field. The etiology of autoimmune disease is usually related to immunity regulatory genetic factors which are altered their expression through various mechanisms [101]. To widely rapidly sensing the expression of genes, biosensors with DNA or aptamers grafted the surface of substrates are the appropriate candidate for the detection. For example, Guerrero et al. functionalized a carbon-based electrode with carboxylated- or neutravidin-surfaces; thus, anti-cyclic citrullinated peptide as a biomarker are further immobilized on the functionalized electrode to detect rheumatoid arthritis autoimmune diseases [102]. Then, this electrochemical biosensor provides a high sensitivity (0.8–25 IU/mL) for rheumatoid factors. Compared to the ELISA method, this electrochemical biosensor requires only 25 μL of samples (four times smaller), indicating that the immuno-biosensor possesses a feasibility to achieve rapidly micro-detection.

#### 3.3.4. Biosensors in Cancer Detection

Cancer research has been studies from last past several decades. Traditionally, cancer research focuses the therapy of cancer diseases so that many new efficient therapeutic methods are widely developed. But, in some case, patients with cancer diseases are often discovered at last stage, causing that patients missed the best time of therapy [103]. Therefore, how to rapidly and precisely diagnose or prognose cancer diseases is an important and popular topic for cancer research recently. So far, many biomimetic cancer models have been widely developed for investigating the formation, metastasis, mutation and other mechanism of cancer cells. For example, Kamei and co-worker combined a microfluidic chip, heart, and liver cancer cells as a cancer-on-a-chip model [104]. Via this co-culture model, the effects of drugs in blood circulation loop on metabolism and migration of liver cancer cells could be easily observed. For early detection, label-free biosensors are widely used to provide a rapid easy strategy to fabricate versatile cancer biosensors. Pan et al. developed a dual biomarkers-label chip (i.e., VEGF- and PSA-labeled) for prostate cancers and their circulating tumor cells (Figure 3f) [96]. These biomarkers are designed as thiolated biomarkers for immobilization on a gold nanorods (GNR)-deposited silicon chip. Afterward, the targeting analytes secreted from cancer cells in human serum could be bound on the marker-labeled chip and analyzed by using UV-Vis absorbance within one hour. In addition, Hu and co-workers also developed another type visible signal-amplifiable biosensor for detection. They used a nanoparticle-based chip to capture extracellular vesicle (EV)-associated RNA for overcoming the challenge that low expression EV-associated RNA in cancers at early stage are difficult for detection. By the effect of catalyzed hairpin DNA circuit in cationic polymer nanoparticles, it can trigger the binding of glypican-1 mRNA (pancreatic cancer markers) in serum EV and generate multiple signal outputs within 30 min; the technique enables to distinguish patients with pancreatic cancer at early- or late-stage. The abovementioned information shows that label-free biosensors with genomic probes play an indispensable role for rapid early detection of cancer diseases (Figure 3g) [97].

### 3.4. Biosensors in Bioimaging Applications

**Figure 4:**
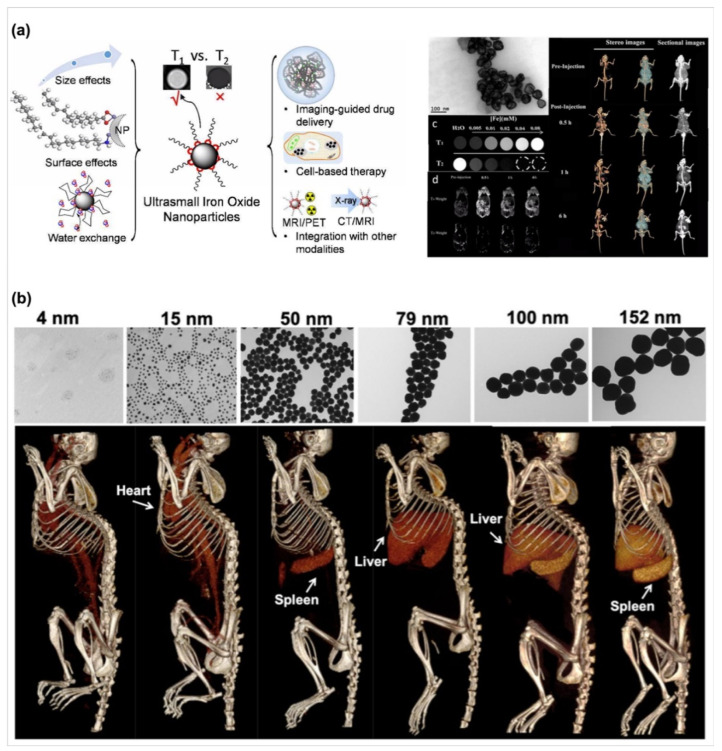
(**a**) Schematic illustrating ultra small iron oxide nanoparticles as T1 magnetic resonance imaging (MRI) contrast agents and possible integration with positron emission tomography (PET) and computed tomography (CT). The ultra small iron oxide nanoparticles further modified with folic acid enable to significant enhance the labels in the tumor and kidney regions in dual CT scan/MRI images, confirming that the nanoparticles could be used as contrast agents for both CT scan/MRI imaging. (Reprinted with permission from [108] Copyright (2018) Royal Society of Chemistry.) (**b**) The TEM images of gold nanoparticles with different particle sizes. Representative 3D CT images displayed at a window level of 1090 HU and window width of 930 HU, indicating that controlling the size of nanoparticles can enhance the image contrast at various organs. (Reprinted with permission from [109] Copyright (2019) Springer Nature Limited.).

Next, in addition to the in vitro diagnosis applications, biosensors offer an in vivo targeting property for enhancing the sensing ability in bioimaging applications because biosensors possess a rapid precise labeling function. Based on this concept, high surface areas, modifiable properties, and small size characterization endow nanomaterials with a versatile type for use which are a candidate for bioimaging [105]. According to types of diagnosis machines such as magnetic resonance imaging (MRI), computer topography imaging (CT imaging), fluorescent microscope images, several types of nanomaterials including silicas, polymers, carbon dots and others have been designed to use for different purposes [105]. Palantavida et al. designed mesoporous silica nanoparticles encapsulated with a near-infrared (NIR) fluorescent dye, LS277, as a breast cancer cell targeting molecule [106]. Compared with the use of only LS277, the mesoporous silica nanoparticle provides the cell labeling bioimages with five times resolution than that in use only LS277 under the short exposure time. Moreover, Bao and co-workers also utilized a NIR-trigger technique to develop carbon dot-based nanoparticles [107]. Via the synthesis of carbon dots with citric acid, urea, and DMSO, a S, N-doped carbon dot with NIR fluorescence could be generated and be excluded from major organs such as liver or kidney quickly after 24-h post intravenous injection. Interestingly, after intravenous injection, the NIR fluorescent images showed that S, N-doped carbon dots significantly accumulate in tumors after three hours, indicating that S, N-doped carbon dots enable the tumor labeling of a compound. However, the absorption and scatter of under subcutaneous tissues and tissue-endogenous fluorescence limit the transmission distance (around 5 mm) of NIR fluorescent molecules. To obtain deeper bioimages, Bao et al. and Dong et al. developed a magnetic iron oxide nanoparticle and a gold nanoparticle for bioimaging in MRI and CT techniques [108,109] respectively. Both results from their studies contribute to an important information that the nanoparticles offer a function as contrast medium for use in MRI and CT techniques. By regulating the size of magnetic iron oxide or gold nanoparticles, the nanoparticle can enhance the image contrast at various organs (Figure 4). For example, the size of magnetic iron oxide and gold nanoparticles around 4 nm could enhance the T1 image contrasts and the images of tissues with the high attenuation change in MRI and CT images, respectively [108,109]. Furthermore, through chemical molecule modification such as folic acid on the surface of nanoparticles endows these nanoparticles with a function as a tumor contrast medium [108]. Therefore, a variety of types of nanoparticles as biosensors offer potential use for bioimaging applications.

## 4. Challenges and Future Perspectives

Biosensors contribute to selective, sensitive, and rapid tools for the disease diagnosis in tissue engineering applications. Compared to standard ELISA analytical technique, biosensors provide a strategy to real-time and on-site monitor micro biophysiological signals via the combination of biological, chemical, and physical technologies. Although biosensors have well developed in the past decade, there are still some challenges needed to be improved. The major challenges of biosensors are the scale-up process and the long-term stability of commercial products. The current biosensors are usually shown as a prototype in a research laboratory or academic departments. Therefore, as commercial products, the scale-up technology is important to rapidly mass-produce biosensors with good quality from laboratory level to industry level. Exactly precisely translating academic results in a commercially viable industry process is difficult and high-cost so that time-consuming and limited funding source may strict the development of scale-up process in academic research and industrial design. Furthermore, from industry level to retail level, commercial biosensors usually require a long time until they are sold to a user. However, some biosensors are designed by immobilizing antigens of grow factors, proteins, or nucleus acids whose short half-time might cause a short expiration date and require a strict storage environment for preventing to degradation of antigen. Therefore, these types of biosensors face a stability issue for successfully sensing after long-term storage. Additionally, biosensors allow for recording extremely weak signals from tiny samples under a quiet and low noise environment. However, there are many unpredict and complex matrix existing in a real clinical environment, resulting in that strong background noise and interferences are easily generated to decrease precision of results during the detection process. Moreover, biosensors usually need to connect with controllers, displayers, and other equipment. Therefore, the complex operation and un-friendly interfaces may decrease the willingness of user.

To overcome the abovementioned issues, combination of disciplinary technologies is necessary to develop more high-sensitive and high-speed responsive units for the fabrication of biosensors. For example, nanotechnology endows user to develop materials with various nanostructures such as rod, nanoparticle, pillar and others and then enhance the signal of samples and the sensitivity of transducers via incorporation of these nanomaterials into biosensors; it can facilitate to obtain more precise results in the complex sample and environment [110]. Additionally, though several in vitro cell-based biosensors show real-time and continuous monitoring properties, small lightweight biosensors such as paper-based biosensors provide an easy strategy to directly observe the results that dissolve the complex operation issues. At meanwhile, these lightweight biosensors could improve the complex produced processes in industry and further achieve the feasibility of scale-up from laboratory level to industry level. Therefore, the current trend of biosensors in the tissue engineering field is the development of small, lightweight, and flexible biosensors. This type of biosensor brings a new opening view to incorporate biosensors in wearable devices. For example, tear-based biosensing technology has been used to generate contact-lens biosensors. Through the detection of tear, this biosensor could be used for continuous monitoring of diabetes [111]. This information shows that wearable biosensors with a potential as a non-invasive measurement method to directly obtain the health information from human by detecting sweat, saliva, tear, and other body fluids. Furthermore, the wearable biosensors combined with apps and smart phones bring a new sight to develop the next generation biosensors to use for real-time continuous monitoring, diagnosing, or prognosing diseases. Therefore, through the publication of this review, we hope to provide scientists who work in biology, chemistry, and physics with the information to understand how to design the desired biosensors related to diseases for various applications in tissue engineering. Thus, more worthwhile potential functions of biosensors for use in academic research and clinical applications can be discovered.

## References

[1] Bhalla N., Jolly P., Formisano N., Estrela P. (2016). Introduction to biosensors. Essays Biochem..

[2] Clark L.C., Lyons C. (1962). Electrode Systems for Continuous Monitoring in Cardiovascular Surgery. Ann. N. Y. Acad. Sci..

[3] Turner A.P.F. (2013). Biosensors: Sense and sensibility. Chem. Soc. Rev..

[4] Vigneshvar S., Sudhakumari C.C., Senthilkumaran B., Prakash H. (2016). Recent Advances in Biosensor Technology for Potential Applications—An Overview. Front Bioeng. Biotech..

[5] Zhang W., Li X., Zou R., Wu H., Shi H., Yu S., Liu Y. (2015). Multifunctional glucose biosensors from Fe(3)O(4) nanoparticles modified chitosan/graphene nanocomposites. Sci. Rep..

[6] Zhang W.J., Chen C.P., Yang D.X., Dong G.X., Jia S.J., Zhao B.X., Yan L., Yao Q.Q., Sunna A., Liu Y. (2016). Optical Biosensors Based on Nitrogen-Doped Graphene Functionalized with Magnetic Nanoparticles. Adv. Mater. Interfaces.

[7] Bousse L. (1996). Whole cell biosensors. Sensor. Actuators B-Chem..

[8] Ma C., Fan R., Ahmad H., Shi Q., Comin-Anduix B., Chodon T., Koya R.C., Liu C.-C., Kwong G.A., Radu C.G. (2011). A clinical microchip for evaluation of single immune cells reveals high functional heterogeneity in phenotypically similar T cells. Nat. Med..

[9] Zhu H., Stybayeva G., Macal M., Ramanculov E., George M.D., Dandekar S., Revzin A. (2008). A microdevice for multiplexed detection of T-cell-secreted cytokines. Lab Chip.

[10] Du H., Strohsahl C.M., Camera J., Miller B.L., Krauss T.D. (2005). Sensitivity and Specificity of Metal Surface-Immobilized “Molecular Beacon” Biosensors. J. Amn. Chem. Soc..

[11] Contreras-Naranjo J.E., Aguilar O. (2019). Suppressing Non-Specific Binding of Proteins onto Electrode Surfaces in the Development of Electrochemical Immunosensors. Biosensors (Basel).

[12] Arya S.K., Park M.K. (2014). 4-Fluoro-3-nitrophenyl grafted gold electrode based platform for label free electrochemical detection of interleukin-2 protein. Biosens. Bioelectron..

[13] Chen Z., Sun M., Luo F., Xu K., Lin Z., Zhang L. (2018). Stimulus-response click chemistry based aptamer-functionalized mesoporous silica nanoparticles for fluorescence detection of thrombin. Talanta.

[14] Wu D., Liu Y., Wang Y., Hu L., Ma H., Wang G., Wie Q. (2016). Label-free Electrochemiluminescent Immunosensor for Detection of Prostate Specific Antigen based on Aminated Graphene Quantum Dots and Carboxyl Graphene Quantum Dots. Sci. Rep..

[15] Liu Q., Wu C., Cai H., Hu N., Zhou J., Wang P. (2014). Cell-based biosensors and their application in biomedicine. Chem. Rev..

[16] Gui Q., Lawson T., Shan S., Yan L., Liu Y. (2017). The Application of Whole Cell-Based Biosensors for Use in Environmental Analysis and in Medical Diagnostics. Sensors (Basel).

[17] Raut N., O’Connor G., Pasini P., Daunert S. (2012). Engineered cells as biosensing systems in biomedical analysis. Anal. Bioanal. Chem..

[18] Fang Y., Ferrie A.M., Fontaine N.H., Mauro J., Balakrishnan J. (2006). Resonant Waveguide Grating Biosensor for Living Cell Sensing. Biophys. J..

[19] Bavli D., Prill S., Ezra E., Levy G., Cohen M., Vinken M., Vanfleteren J., Jaeger M., Nahmias Y. (2016). Real-time monitoring of metabolic function in liver-on-chip microdevices tracks the dynamics of mitochondrial dysfunction. Proc. Natl. Acad. Sci. USA.

[20] Hammarback J.A., Palm S.L., Furcht L.T., Letourneau P.C. (1985). Guidance of neurite outgrowth by pathways of substratum-adsorbed laminin. J. Neurosci. Res..

[21] Derkus B. (2016). Applying the miniaturization technologies for biosensor design. Biosens. Bioelectron..

[22] Mir M., Dondapati S.K., Duarte M.V., Chatzichristidi M., Misiakos K., Petrou P., Kakabakos S.E., Argitis P., Katakis I. (2010). Electrochemical biosensor microarray functionalized by means of biomolecule friendly photolithography. Biosens. Bioelectron..

[23] Pires N.M.M., Dong T., Yang Z. (2019). A fluorimetric nitrite biosensor with polythienothiophene-fullerene thin film detectors for on-site water monitoring. Analyst.

[24] Diakoumakos C.D., Douvas A., Raptis I., Kakabakos S., Dimotikalli D., Terzoudi G., Argitis P. (2002). Dilute aqueous base developable resists for environmentally friendly and biocompatible processes. Microelectron. Eng..

[25] Petrou P.S., Chatzichristidi M., Douvas A.M., Argitis P., Misiakos K., Kakabakos S.E. (2007). A biomolecule friendly photolithographic process for fabrication of protein microarrays on polymeric films coated on silicon chips. Biosens. Bioelectron..

[26] Tran K.T.M., Nguyen T.D. (2017). Lithography-based methods to manufacture biomaterials at small scales. J. Sci. Adv. Mater. Dev..

[27] Ganter P., Lotsch B.V. (2017). Photocatalytic Nanosheet Lithography: Photolithography based on Organically Modified Photoactive 2D Nanosheets. Angew. Chem. Int..

[28] Qin D., Xia Y., Whitesides G.M. (2010). Soft lithography for micro- and nanoscale patterning. Nat. Protoc..

[29] Sharma S., Huang Z., Rogers M., Boutelle M., Cass A.E.G. (2016). Evaluation of a minimally invasive glucose biosensor for continuous tissue monitoring. Anal. Bioanal. Chem..

[30] Kim M., Kim T., Kim D.S., Chung W.K. (2015). Curved Microneedle Array-Based sEMG Electrode for Robust Long-Term Measurements and High Selectivity. Sensors (Basel).

[31] Shin S.R., Farzad R., Tamayol A., Manoharan V., Mostafalu P., Zhang Y.S., Akbari M., Jung S.M., Kim D., Comotto M. (2016). A Bioactive Carbon Nanotube-Based Ink for Printing 2D and 3D Flexible Electronics. Adv. Mater..

[32] Manzanares Palenzuela C.L., Novotny F., Krupicka P., Sofer Z., Pumera M. (2018). 3D-Printed Graphene/Polylactic Acid Electrodes Promise High Sensitivity in Electroanalysis. Anal. Chem..

[33] Truby R.L., Wehner M., Grosskopf A.K., Vogt D.M., Uzel S.G.M., Wood R.J., Lewis J.A. (2018). Soft Somatosensitive Actuators via Embedded 3D Printing. Adv. Mater..

[34] Zheng Y., Wang L., Lu L., Wang Q., Benicewicz B.C. (2017). pH and Thermal Dual-Responsive Nanoparticles for Controlled Drug Delivery with High Loading Content. ACS Omega.

[35] Zhou H., Qin W., Yu Q., Cheng H., Yu X., Wu H. (2019). Transfer Printing and its Applications in Flexible Electronic Devices. Nanomaterials (Basel).

[36] Xu L., Shyu T.C., Kotov N.A. (2017). Origami and Kirigami Nanocomposites. ACS Nano.

[37] Sim K., Chen S., Li Y., Kammoun M., Peng Y., Xu M., Gao Y., Song J., Zhang Y., Ardebili H. (2015). High Fidelity Tape Transfer Printing Based On Chemically Induced Adhesive Strength Modulation. Sci. Rep..

[38] Rafsanjani A., Zhang Y., Liu B., Rubinstein S.M., Bertoldi K. (2018). Kirigami skins make a simple soft actuator crawl. Sci. Robot..

[39] Evke E.E., Meli D., Shtein M. (2019). Developable Rotationally Symmetric Kirigami-Based Structures as Sensor Platforms. Adv. Mater. Technol..

[40] Hardin J.O., Ober T.J., Valentine A.D., Lewis J.A. (2015). Microfluidic Printheads for Multimaterial 3D Printing of Viscoelastic Inks. Adv. Mater..

[41] Chang C.C., Boland E.D., Williams S.K., Hoying J.B. (2011). Direct-write bioprinting three-dimensional biohybrid systems for future regenerative therapies. J. Biomed. Mater. Res. B.

[42] Duan B., Hockaday L.A., Kang K.H., Butcher J.T. (2013). 3D bioprinting of heterogeneous aortic valve conduits with alginate/gelatin hydrogels. J. Biomed. Mater. Res. A.

[43] Schuurman W., Khristov V., Pot M.W., van Weeren P.R., Dhert W.J., Malda J. (2011). Bioprinting of hybrid tissue constructs with tailorable mechanical properties. Biofabrication.

[44] Xu T., Zhao W., Zhu J.M., Albanna M.Z., Yoo J.J., Atala A. (2013). Complex heterogeneous tissue constructs containing multiple cell types prepared by inkjet printing technology. Biomaterials.

[45] Cui X., Breitenkamp K., Finn M.G., Lotz M., D’Lima D.D. (2012). Direct human cartilage repair using three-dimensional bioprinting technology. Tissue Eng. Part A.

[46] Cui X., Boland T., D’Lima D.D., Lotz M.K. (2012). Thermal inkjet printing in tissue engineering and regenerative medicine. Recent Patents Drug Deliv. Formul..

[47] Christensen K., Xu C., Chai W., Zhang Z., Fu J., Huang Y. (2015). Freeform inkjet printing of cellular structures with bifurcations. Biotechnol. Bioen..

[48] Whatley B.R., Li X., Zhang N., Wen X. (2014). Magnetic-directed patterning of cell spheroids. J. Biomed. Mater. Res. A.

[49] Koch L., Gruene M., Unger C., Chichkov B. (2013). Laser assisted cell printing. Curr. Pharm. Biotechnol..

[50] Guillotin B., Souquet A., Catros S., Duocastella M., Pippenger B., Bellance S., Bareille R., Remy M., Bordenave L., Amedee J. (2010). Laser assisted bioprinting of engineered tissue with high cell density and microscale organization. Biomaterials.

[51] Michael S., Sorg H., Peck C.T., Koch L., Deiwick A., Chichkov B., Vogt P.M., Reimers K. (2013). Tissue engineered skin substitutes created by laser-assisted bioprinting form skin-like structures in the dorsal skin fold chamber in mice. PLoS ONE.

[52] Sharafeldin M., Jones A., Rusling J.F. (2018). 3D-Printed Biosensor Arrays for Medical Diagnostics. Micromachines (Basel).

[53] Holzinger M., Le Goff A., Cosnier S. (2014). Nanomaterials for biosensing applications: A review. Front. Chem..

[54] Farka Z., Jurik T., Kovar D., Trnkova L., Skladal P. (2017). Nanoparticle-Based Immunochemical Biosensors and Assays: Recent Advances and Challenges. Chem. Rev..

[55] Kabe Y., Sakamoto S., Hatakeyama M., Yamaguchi Y., Suematsu M., Itonaga M., Handa H. (2019). Application of high-performance magnetic nanobeads to biological sensing devices. Anal. Bioanal. Chem..

[56] Min J.H., Woo M.K., Yoon H.Y., Jang J.W., Wu J.H., Lim C.S., Kim Y.K. (2014). Isolation of DNA using magnetic nanoparticles coated with dimercaptosuccinic acid. Anal. Biochem..

[57] Langer R., Vacanti J.P. (1993). Tissue engineering. Science.

[58] Hasan A., Nurunnabi M., Morshed M., Paul A., Polini A., Kuila T., Al Hariri M., Lee Y.-K., Jaffa A.A. (2014). Recent Advances in Application of Biosensors in Tissue Engineering. BioMed. Res. Int..

[59] Kim Y.E., Chen J., Chan J.R., Langen R. (2010). Engineering a polarity-sensitive biosensor for time-lapse imaging of apoptotic processes and degeneration. Nat. Methods.

[60] Chowdhury R.A., Tzortzis K.N., Dupont E., Selvadurai S., Perbellini F., Cantwell C.D., Ng F.S., Simon A.R., Terracciano C.M., Peters N.S. (2018). Concurrent micro- to macro-cardiac electrophysiology in myocyte cultures and human heart slices. Sci. Rep..

[61] Knöll R., Hoshijima M., Hoffman H.M., Person V., Lorenzen-Schmidt I., Bang M.-L., Hayashi T., Shiga N., Yasukawa H., Schaper W. (2002). The Cardiac Mechanical Stretch Sensor Machinery Involves a Z Disc Complex that Is Defective in a Subset of Human Dilated Cardiomyopathy. Cell.

[62] Caluori G., Pribyl J., Pesl M., Jelinkova S., Rotrekl V., Skladal P., Raiteri R. (2019). Non-invasive electromechanical cell-based biosensors for improved investigation of 3D cardiac models. Biosens. Bioelectron..

[63] Son K.J., Gheibi P., Stybayeva G., Rahimian A., Revzin A. (2017). Detecting cell-secreted growth factors in microfluidic devices using bead-based biosensors. Microsyst. Nanoeng..

[64] Shen R., Liu P., Zhang Y., Yu Z., Chen X., Zhou L., Nie B., Żaczek A., Chen J., Liu J. (2018). Sensitive Detection of Single-Cell Secreted H2O2 by Integrating a Microfluidic Droplet Sensor and Au Nanoclusters. Anal. Chem..

[65] Wang Y., Hu S. (2006). A novel nitric oxide biosensor based on electropolymerization poly(toluidine blue) film electrode and its application to nitric oxide released in liver homogenate. Biosens. Bioelectron..

[66] Visser C.W., Kamperman T., Karbaat L.P., Lohse D., Karperien M. (2018). In-air microfluidics enables rapid fabrication of emulsions, suspensions, and 3D modular (bio)materials. Sci. Adv..

[67] Kato K., Ishimuro T., Arima Y., Hirata I., Iwata H. (2007). High-Throughput Immunophenotyping by Surface Plasmon Resonance Imaging. Anal. Chem..

[68] Kuo Y.-C., Ho J.H., Yen T.-J., Chen H.-F., Lee O.K.-S. (2011). Development of a surface plasmon resonance biosensor for real-time detection of osteogenic differentiation in live mesenchymal stem cells. PLoS ONE.

[69] Andersson H., Steel D., Asp J., Dahlenborg K., Jonsson M., Jeppsson A., Lindahl A., Kågedal B., Sartipy P., Mandenius C.-F. (2010). Assaying cardiac biomarkers for toxicity testing using biosensing and cardiomyocytes derived from human embryonic stem cells. J. Biotech..

[70] Fathi F., Rezabakhsh A., Rahbarghazi R., Rashidi M.-R. (2017). Early-stage detection of VE-cadherin during endothelial differentiation of human mesenchymal stem cells using SPR biosensor. Biosens. Bioelectron..

[71] Rutten M.J., Laraway B., Gregory C.R., Xie H., Renken C., Keese C., Gregory K.W. (2015). Rapid assay of stem cell functionality and potency using electric cell-substrate impedance sensing. Stem Cell Res. Ther..

[72] Price D.T., Rahman A.R., Bhansali S. (2009). Design rule for optimization of microelectrodes used in electric cell-substrate impedance sensing (ECIS). Biosens. Bioelectron..

[73] Wegener J., Keese C.R., Giaever I. (2000). Electric cell-substrate impedance sensing (ECIS) as a noninvasive means to monitor the kinetics of cell spreading to artificial surfaces. Exp. Cell Res..

[74] Szulcek R., Bogaard H.J., van Nieuw Amerongen G.P. (2014). Electric cell-substrate impedance sensing for the quantification of endothelial proliferation, barrier function, and motility. J. Vis. Exp..

[75] Xiao C., Luong J.H.T. (2003). On-Line Monitoring of Cell Growth and Cytotoxicity Using Electric Cell-Substrate Impedance Sensing (ECIS). Biotech. Prog..

[76] Koo Y., Yun Y. (2016). Effects of polydeoxyribonucleotides (PDRN) on wound healing: Electric cell-substrate impedance sensing (ECIS). Mater. Sci. Eng. C.

[77] Anchan A., Kalogirou-Baldwin P., Johnson R., Kho D.T., Joseph W., Hucklesby J., Finlay G.J., O’Carroll S.J., Angel C.E., Graham E.S. (2019). Real-Time Measurement of Melanoma Cell-Mediated Human Brain Endothelial Barrier Disruption Using Electric Cell-Substrate Impedance Sensing Technology. Biosensors.

[78] Bagnaninchi P.O., Drummond N. (2011). Real-time label-free monitoring of adipose-derived stem cell differentiation with electric cell-substrate impedance sensing. Proc.Natl. Acad.Sci. USA.

[79] Fleischer S., Jahnke H.-G., Fritsche E., Girard M., Robitzki A.A. (2019). Comprehensive human stem cell differentiation in a 2D and 3D mode to cardiomyocytes for long-term cultivation and multiparametric monitoring on a multimodal microelectrode array setup. Biosens. Bioelectron..

[80] Kalmykov A., Huang C., Bliley J., Shiwarski D., Tashman J., Abdullah A., Rastogi S.K., Shukla S., Mataev E., Feinberg A.W. (2019). Organ-on-e-chip: Three-dimensional self-rolled biosensor array for electrical interrogations of human electrogenic spheroids. Sci. Adv..

[81] Raknim P., Lan K.C. (2016). Gait Monitoring for Early Neurological Disorder Detection Using Sensors in a Smartphone: Validation and a Case Study of Parkinsonism. Telemed. J. e-Health.

[82] Seymour J.P., Wu F., Wise K.D., Yoon E. (2017). State-of-the-art MEMS and microsystem tools for brain research. Microsyst. Nanoeng..

[83] Chiappalone M., Vato A., Tedesco M., Marcoli M., Davide F., Martinoia S. (2003). Networks of neurons coupled to microelectrode arrays: A neuronal sensory system for pharmacological applications. Biosens. Bioelectron..

[84] Lourenço C.F., Ledo A., Gerhardt G.A., Laranjinha J., Barbosa R.M. (2017). Neurometabolic and electrophysiological changes during cortical spreading depolarization: Multimodal approach based on a lactate-glucose dual microbiosensor arrays. Sci. Rep..

[85] Liu Y.-C., Lee I.C., Lei K.F. (2018). Toward the Development of an Artificial Brain on a Micropatterned and Material-Regulated Biochip by Guiding and Promoting the Differentiation and Neurite Outgrowth of Neural Stem/Progenitor Cells. ACS Appl. Mater. Inter..

[86] Liu L., Xiao X., Lei K.F., Huang C.-H. (2015). Quantitative Impedimetric Monitoring of Cell Migration Under the Stimulation of Cytokine or Anti-Cancer Drug in a Microfluidic Chip. Biomicrofluidics.

[87] Lei K.F., Tseng H.-P., Lee C.-Y., Tsang N.-M. (2016). Quantitative Study of Cell Invasion Process under Extracellular Stimulation of Cytokine in a Microfluidic Device. Sci. Rep..

[88] Lei K.F., Lin B.-Y., Tsang N.-M. (2017). Real-time and Label-Free Impedimetric Analysis of the Formation and Drug Testing of Tumor Spheroids Formed via the Liquid Overlay Technique. RSC Adv..

[89] Vomero M., Castagnola E., Ciarpella F., Maggiolini E., Goshi N., Zucchini E., Carli S., Fadiga L., Kassegne S., Ricci D. (2017). Highly Stable Glassy Carbon Interfaces for Long-Term Neural Stimulation and Low-Noise Recording of Brain Activity. Sci. Rep..

[90] Xie C., Liu J., Fu T.M., Dai X., Zhou W., Lieber C.M. (2015). Three-dimensional macroporous nanoelectronic networks as minimally invasive brain probes. Nat. Mater..

[91] Li X., Soler M., Özdemir C.I., Belushkin A., Yesilköy F., Altug H. (2017). Plasmonic nanohole array biosensor for label-free and real-time analysis of live cell secretion. Lab Chip.

[92] Li S.S., Lin C.W., Wei K.C., Huang C.Y., Hsu P.H., Liu H.L., Lu Y.J., Lin S.C., Yang H.W., Ma C.C. (2016). Non-invasive screening for early Alzheimer’s disease diagnosis by a sensitively immunomagnetic biosensor. Sci. Rep..

[93] Yang X., Li H., Zhao X., Liao W., Zhang C.X., Yang Z. (2020). A novel, label-free liquid crystal biosensor for Parkinson’s disease related alpha-synuclein. Chem. Commun..

[94] Lee S.P., Ha G., Wright D.E., Ma Y., Sen-Gupta E., Haubrich N.R., Branche P.C., Li W., Huppert G.L., Johnson M. (2018). Highly flexible, wearable, and disposable cardiac biosensors for remote and ambulatory monitoring. NPJ Digit. Med..

[95] Liu H., Bolonduro O.A., Hu N., Ju J., Rao A.A., Duffy B.M., Huang Z., Black L.D., Timko B.P. (2020). Heart-on-a-Chip Model with Integrated Extra- and Intracellular Bioelectronics for Monitoring Cardiac Electrophysiology under Acute Hypoxia. Nano Lett..

[96] Pan L.H., Pang S.T., Fang P.Y., Chuang C.K., Yang H.W. (2017). Label-Free Biochips for Accurate Detection of Prostate Cancer in the Clinic: Dual Biomarkers and Circulating Tumor Cells. Theranostics.

[97] Hu J., Sheng Y., Kwak K.J., Shi J., Yu B., Lee L.J. (2017). A signal-amplifiable biochip quantifies extracellular vesicle-associated RNAs for early cancer detection. Nat. Commun..

[98] Duranteau J., Chandel N.S., Kulisz A., Shao Z., Schumacker P.T. (1998). Intracellular signaling by reactive oxygen species during hypoxia in cardiomyocytes. J. Biol. Chem..

[99] Dutta S., Minchole A., Quinn T.A., Rodriguez B. (2017). Electrophysiological properties of computational human ventricular cell action potential models under acute ischemic conditions. Prog. Biophys. Mol. Biol..

[100] Feiner R., Fleischer S., Shapira A., Kalish O., Dvir T. (2018). Multifunctional degradable electronic scaffolds for cardiac tissue engineering. J. Control Release.

[101] Giana G., Romano E., Porfirio M.C., D’Ambrosio R., Giovinazzo S., Troianiello M., Barlocci E., Travaglini D., Granstrem O., Pascale E. (2015). Detection of auto-antibodies to DAT in the serum: Interactions with DAT genotype and psycho-stimulant therapy for ADHD. J. Neuroimmunol..

[102] Guerrero S., Sanchez-Tirado E., Martinez-Garcia G., Gonzalez-Cortes A., Yanez-Sedeno P., Pingarron J.M. (2020). Electrochemical biosensor for the simultaneous determination of rheumatoid factor and anti-cyclic citrullinated peptide antibodies in human serum. Analyst.

[103] Fayanju O.M., Jeffe D.B., Elmore L., Ksiazek D.N., Margenthaler J.A. (2013). Patient and process factors associated with late-stage breast cancer diagnosis in Safety-Net patients: A pilot prospective study. Ann. Surg. Oncol..

[104] Kamei K.-i., Yoshiki K., Hirai Y., Ito S., Satoh J., Oka A., Tsuchiya T., Chenad Y., Tabatab O. (2017). Integrated heart/cancer on a chip to reproduce the side effects of anti-cancer drugs in vitro. RSC Adv..

[105] Wolfbeis O.S. (2015). An overview of nanoparticles commonly used in fluorescent bioimaging. Chem. Soc. Rev..

[106] Palantavida S., Tang R., Sudlow G.P., Akers W.J., Achilefu S., Sokolov I. (2014). Ultrabright NIR fluorescent mesoporous silica nanoparticles. J. Mater. Chem. B.

[107] Bao X., Yuan Y., Chen J., Zhang B., Li D., Zhou D., Jing P., Xu G., Wang Y., Hola K. (2018). In vivo theranostics with near-infrared-emitting carbon dots-highly efficient photothermal therapy based on passive targeting after intravenous administration. Light Sci. Appl..

[108] Bao Y., Sherwood J.A., Sun Z. (2020). Magnetic iron oxide nanoparticles as T1 contrast agents for magnetic resonance imaging. J. Mater. Chem. C.

[109] Dong Y.C., Hajfathalian M., Maidment P.S.N., Hsu J.C., Naha P.C., Si-Mohamed S., Breuilly M., Kim J., Chhour P., Douek P. (2019). Effect of Gold Nanoparticle Size on Their Properties as Contrast Agents for Computed Tomography. Sci. Rep..

[110] Wang X., Lu X., Chen J. (2014). Development of biosensor technologies for analysis of environmental contaminants. Trends Environ. Anal. Chem..

[111] Tseng R.C., Chen C.C., Hsu S.M., Chuang H.S. (2018). Contact-Lens Biosensors. Sensors (Basel).

